# Use of Immersive Virtual Reality Spaces to Engage Adolescent and Young Adult Patients With Cancer in Therapist-Guided Support Groups: Protocol for a Pre-Post Study

**DOI:** 10.2196/48761

**Published:** 2023-11-09

**Authors:** Asher Marks, Amanda Garbatini, Kimberly Hieftje, Vidya Puthenpura, Veronica Weser, Claudia-Santi F Fernandes

**Affiliations:** 1 Section of Pediatric Hematology/Oncology Department of Pediatrics Yale School of Medicine New Haven, CT United States; 2 Department of Social Work Yale New Haven Hospital New Haven, CT United States; 3 Department of Pediatrics Yale School of Medicine New Haven, CT United States; 4 Department of General Internal Medicine Yale School of Medicine New Haven, CT United States; 5 Yale Child Study Center Yale School of Medicine New Haven, CT United States

**Keywords:** cancer, virtual reality, support groups, peer support, adolescent, young adult, resilience, adolescents and young adults, oncology, therapist-guided support, social isolation, support system, psychosocial support, barrier, quality of life

## Abstract

**Background:**

For adolescents and young adults, a cancer diagnoses can magnify feelings of social isolation at an inherently vulnerable developmental stage. Prior studies have highlighted the importance of peer groups during cancer treatment. Support groups help foster connection and resilience, but patients find in-person participation difficult due to a variety of factors. Additionally, physical changes brought on by cancer makes these patients hesitant to meet in person. The COVID-19 pandemic magnified these difficulties. Virtual reality (VR) allows for the creation of a therapist-curated, computer-generated social space that potentially enables support groups for this population.

**Objective:**

This protocol describes a pilot study examining the efficacy, feasibility, and acceptability of a social VR support group intervention for adolescent and young adult patients with cancer.

**Methods:**

We approached 20 participants aged 17-20 years, and 16 agreed to participate. Moreover, 1 participant dropped out due to hospitalization. Participants attended virtual, professionally facilitated support groups using Meta Quest VR headsets. The groups consisted of 4 participants and 1 facilitator, amounting to a total of 22 individual sessions. Each session lasted 45-60 minutes and took place weekly for 4-6 weeks. The primary aim of this study was to collect quantitative and qualitative data on the feasibility and acceptability of the intervention. Feasibility was measured through session participation rates and overall retention rates. The acceptability of the intervention was explored through brief in-person interviews with participants at the end of the final intervention session. The secondary aim of this study was to collect data on the preliminary efficacy of the intervention in decreasing symptoms of participant depression and anxiety and increasing positive affect and resiliency.

**Results:**

In total, 15 patients aged 17-20 years participated in 22 sessions between November 5, 2019, and July 8, 2021. The median age was 19 (IQR 17-20) years. Overall, 10 (62%) participants identified as male, 5 (31%) as female, and 1 (6%) as transgender female. Furthermore, 5 (31%) participants identified as Hispanic, 1 (6%) identified as non-Hispanic Asian, 3 (19%) identified as non-Hispanic Black, 6 (38%) identified as non-Hispanic White, and 1 (6%) identified as other race or ethnicity. Hematologic malignancies or bone marrow failure was the most common diagnosis (8/16, 50%). The mean attendance rate was 72.8% (SD 25.7%) and retention was 86.7% (SD 0.35%). Moreover, 45% (10/22) of sessions had to be postponed by a week or more due to unexpected participant scheduling issues.

**Conclusions:**

The use of VR to deliver psychosocial support for adolescents and young adults with cancer may reduce common barriers associated with attending in-person peer support groups while improving quality-of-life measures. The data from this study will inform future studies focused on conducting VR support groups in other rare disease populations, including older adults with cancer.

**International Registered Report Identifier (IRRID):**

DERR1-10.2196/48761

## Introduction

### Background

Approximately 90,000 adolescents and young adults were diagnosed with cancer in 2020, with around 9000 having lost their lives [1]. In the group of patients aged 15-39 years, a cancer diagnosis brings unique medical and psychosocial needs. Complications of treatment are both medical and psychosocial and include infection, fatigue, hair loss, mouth sores, anxiety, infertility, depression, boredom, and isolation. Feelings of social isolation, depression, and anxiety are magnified in this group as their life experiences violently diverge from those of their peers. Support from caregivers and interactions with adolescents and young adults going through a similar experience improves the quality of life for this population [2].

One of the most requested and effective psychosocial interventions for this population are peer support groups, whether in person or via videoconference [3,4]. These groups give adolescents and young adults with cancer the opportunity to shed the defining attribute of their cancer diagnoses and engage with peers who have had similar experiences while finding true empathy among peers. Despite the clear benefits of these groups and patients’ desire to participate in them, personal communications with various adolescents and young adult cancer centers and personal experience in attempting to organize these groups have proven the difficulty of assuring in-person attendance. The reason for this is multifold, including active immunosuppression; prolonged hospitalizations; geographic constraints; and patients’ other responsibilities to their schools, families, and jobs. In addition, the inherent physical changes brought on by an adolescents and young adult cancer diagnoses make these patients less willing to meet in person or show themselves on a screen.

### Potential Technologic Approaches to Solve the Problem of Poor Adherence

Although support groups have traditionally been conducted through real time face-to-face gatherings, a lack of access due to distance, time, and transportation has long been cited as one of the most prominent barriers to support group participation and adherence [5]. For individuals with long-term health conditions, such as cancer, these barriers are further amplified [6]. The past decade has seen the steady development of virtual support groups that use the communication technology of virtual worlds to overcome the logistical barriers presented by face-to-face meetings [7,8]. In addition to minimizing logistical barriers, virtual support groups offer further affordances including anonymity, which may facilitate disclosure, autonomy, and help-seeking [9], whereby users can control how much they share and with whom. In the case of persistent online worlds including social media, the asynchrony of participation allows individuals to access the service whenever they choose [10]. Although the COVID-19 pandemic propelled mainstream acceptance of videoconferencing software such as Zoom (Zoom Video Communications) as a convenient tool for remote social connection, the use of live videoconferencing for support groups is not well defined in the literature, although initial reports of its use during the pandemic show it has promise as a solution to the logistical barriers to attendance [11].

Social virtual reality (VR) is an emerging technology in which multiple users can interact with one another in a shared, immersive 3D virtual environment [12]. Compared to other forms of virtual interaction that mainly support screen-mediated communication (including videoconferencing, social media, and persistent online social worlds), social VR is characterized by full-body tracked avatars. These avatars enable real-time embodied interaction that is similar to face-to-face communication in that it includes verbal *and* nonverbal interactions such as voice, gestures, proxemics, gaze, and facial expression [13]. The opportunity for embodied and immersive experiences mediated through VR technology has the potential to replicate people’s everyday activities and is likely to play a role in forming and maintaining interpersonal relationships. Given that VR can be used from the convenience of one’s own home, or even their hospital bed, support groups offered through social VR settings overcome logistical barriers of physical distance and immunosuppression while still maintaining much of the nuance of nonverbal communication and physicality that are seen as hallmarks of in-person support.

### Previous VR Studies in Adolescents and Young Adults, Cancer, and Telemedicine

Multiple studies have demonstrated the benefits and viability of using VR as an intervention in the pediatric and adolescent and young adult health care setting. In a group of pediatric patients with cancer aged 10-17 years undergoing chemotherapy, it was found that patients’ self-reported symptom distress immediately after chemotherapy was significantly lower when they used VR with 1 of 3 distractive experiences (CD ROM-based scenarios: Magic Carpet, Sherlock Holmes Mystery, and Seventh Guest viewed via a head-mounted display) as compared to a session during which they did not use VR [14]. Another adolescent cancer group showed equally promising effects on pain and discomfort using a VR experience during lumbar puncture in the form of a distracting video viewed via a head-mounted display [15]. In a group of pediatric patients using distractive VR while undergoing chronic wound care, pain and anxiety as measured by patients, caregivers, and nurses was significantly lower during and after dressing changes in the VR group versus the group receiving standard distractions; additionally, the time to complete each dressing change was significantly less in the VR group. The distractive VR came in the form of the Ice Age 2: The Meltdown game. In the game, players control “Sid the Sloth,” who slides down a snowy path while trying to collect acorns and avoid obstacles [16].

Prior studies in adolescents and young adults have illustrated the utility of group-based interventions in this particular age demographic. For example, in a group of 15 adolescents and young adults with type 1 diabetes, hemoglobin A_1C_ improved after participation in peer support groups with a psychologist [17]. More significantly, in the Adolescents and Young Adult Health Outcomes and Patient Experiences study [18], adolescents and young adults with cancer who did not receive, but reported needing support groups, were about 4 and 13 times as likely to report needs for talking about their cancer experience with family and friends and meeting peer survivors, respectively.

Given the proven viability, benefits, and safety of VR in the health care setting, as well as the importance of support groups in the adolescent and young adult population, the combination of these 2 interventions may provide increased psychosocial support for adolescents and young adults with a recent cancer diagnosis.

### Commercially Available Social VR Platforms

Over the past 5 years, social VR apps such as VR Chat, AltspaceVR, Horizon Worlds, and Rec Room have emerged as a few exemplars of social VR platforms where people meet, interact, and socialize in new and more immersive ways using a head-mounted display and hand-tracking hardware. Many social VR platforms can be characterized by customizable avatars and persistent virtual worlds, with some platforms emphasizing opportunities for world customization while others favor social gaming or large-scale events such as concerts. Most platforms provide users with an opportunity to create private rooms or environments that only designated “friends” can access, as well as public worlds or spaces where anyone can interact. These platforms continue to attract more users and improve both in interaction quality, features, and ease of use, with many futurists predicting that VR social interaction will become the next major technological evolution for computer-mediated human communication. Indeed, the social VR market is expected to grow at a 14.1% compound annual growth rate between 2021 and 2027 [19].

While many commercially available social VR apps do exist, this project required a specific platform that was (1) Health Insurance Portability and Accountability Act (HIPAA) compliant, (2) easy to use, (3) contained minimal distractions within the experience, (4) used high-quality spatial audio, and (5) facilitated the expression of subtle body language through player avatars. For this study, we used the Foretell Realities platform, as described within the *Methods* section of this paper.

### Study Aims

The primary aim of this study is to evaluate the feasibility and acceptability of a VR intervention to facilitate support groups for adolescents and young adults with cancer. Intervention feasibility is examined through the number of sessions completed by participants and participant retentions rates at the end of this study. The acceptability of the intervention will be examined through participant interviews conducted at the end of the last VR session and solicited feedback at the end of each individual session.

We hypothesize that our intervention will be feasible as measured by overall participation rates of >50% and retention rates of >50%. These numbers were chosen in the context of historical data showing adolescent and young adult patient participation in clinical trials to fall at less than 20% [20,21] and only 41% of Americans with mental health disorders initiating treatment services, with just 33% engaging in enough services for them to be deemed adequate [22]. Our secondary aim is to collect data on the preliminary impact of the intervention on participants’ depression, anxiety, positive affect, and resilience skills. We hypothesize that participants will report a significant decrease in symptoms of depression and anxiety and an increase in positive affect and resiliency skills as measured by standardized patient report instruments from baseline to immediately after intervention. The results of this aim are beyond the scope of this paper and are currently being reviewed for submission of a subsequent mixed methods analysis.

## Methods

### Ethical Considerations

Formal evaluation and adoption of novel human interventions at major institutes include the need for proper and thoughtful assessment by first, a Protocol Review Committee (PRC) to ensure appropriate scientific rigor and then, an institutional review board (IRB) to ensure participant safety. The formal goal of the PRC is to ensure “assessment of the scientific rationale and merit of a proposed study in addition to protocol design, safety parameters, and biostatistical analysis to determine that high quality and appropriate designs have been incorporated” [23]. In the case of this study, the PRC of review was that of Yale Cancer Center, which consists of voting members from various scientific and epidemiologic disciplines. All procedures were approved first by the PRC and then by the Yale University IRB (ID#2000023701). The participants did not receive any compensation.

### Data Collection

The following personal identifying and medical information were collected: email address, phone number, diagnoses, diagnoses date, end of treatment date (if relevant), treatment modalities, gender identity, race and ethnicity, and insurance status. All data collected from the assessments were entered by participants directly into the secure web-based system, Qualtrics (Qualtrics International Inc), which is Yale’s enterprise-wide data management system or directly onto deidentified paper questionnaires, depending on participant choice. This ensured participant confidence that their data would remain confidential. Written consent was obtained via signature from all the participants before their participation, as detailed in the *Study Population and Study Flow* section.

The confidentiality of assessment data was assured by assigning a unique identifier to each participant once they registered for this study. A document linking this study’s identifiers to the patient’s name was maintained by the principal investigator (PI) and was destroyed upon the completion of data collection. All computer data were password protected and all nondigital data (including consent forms) were stored in locked cabinets.

### Study Design

This pilot study uses a pre-post design to evaluate the feasibility, acceptability, and preliminary efficacy of a social VR support group intervention for adolescent and young adult patients with cancer. The team was led by a team with expertise in adolescent and young adult cancer, onco-psychology, social VR, adolescent development, and clinical trial design and evaluation.

### Study Population and Study Flow

#### Recruitment

Candidates for this study were identified through the Yale University School of Medicine’s Pediatric Hematology and Oncology Department and adolescent and young adult clinic. The Director of the adolescent and young adult clinic is the PI and one of the treating oncologists at Yale New Haven Hospital who has firsthand knowledge of the potential participants, participants’ medical and psychiatric histories, and social situations. The PI consulted with the candidate’s primary treating oncologist, other health care providers, and their psychosocial team to assist in determining if a participant was an appropriate candidate for this study. Candidates were considered eligible to participate if they met the following criteria: (1) between 13 and 30 years of age, (2) actively receiving treatment for cancer or completed treatment for no more than 1 year prior to enrollment, (3) English speaking, and (4) able to don a head-mounted display. Exclusion criteria included (1) not English speaking; (2) the lack of reliable access to the internet; and (3) patients who are prone to motion sickness or are experiencing nausea, vomiting, seizures, or other health problems that the medical team deem them unstable to participate. If all inclusion criteria were met and no exclusion criteria were noted, the PI approached the family or patient for study participation.

If a participant was of age to sign the consent form, consent was obtained directly from the participant. In the case of participants under 18 years of age, youth’s assent was obtained along with legal guardian permission from the participant’s legal guardian. If permission could not be obtained from the patient or their parent or legal guardian, the participant was not enrolled. Consent (or assent and permission) were sought after the research protocol and the risks of participation in this study were fully explained. All participants and participants’ families were encouraged to read the appropriate forms and ask questions about any aspect of this study that was not clear. Participants were reminded that they could withdraw from this study at any time if desired and that the decision to not participate would have no impact on their care or relationship with the physicians or investigators.

#### Assessments

To evaluate the preliminary impact of this study, we collected assessment data at 2 time points: prior to the beginning of the first VR group session (baseline) and immediately after the final session.

To assess participants’ depression, anxiety, and positive affect, we used PROMIS (Patient-Reported Outcomes Measurement Information System) questionnaires. The pediatric PROMIS short forms (version 1.0; Depressive Symptoms 8a July 28, 2016 [8 questions]; Anxiety 8a July 27, 2016 [8 questions]; and Positive Affect 8a July 14, 2016 [8 questions]) were used for participants 17 years of age and younger while the adult short forms (Depression 8a June 26, 2016 [8 questions]; Anxiety 8a June 2, 2016 [8 questions]; and Positive Affect 15a July 18, 2017 [8 questions]) were used for participants 18 years of age or older [24-26]. These surveys have been validated in pediatric and adolescent patients with cancer as well as in children and adolescents in other health care settings [24,27].

To assess resilience, we used the Connor-Davidson Resilience Scale (25 questions), which has been validated in multiple settings, including in young adults and in the outpatient psychiatric context [28,29].

#### Brief In-Person Interviews

We conducted brief in-person interviews with participants at the end of the final VR group session to explore participants’ perceptions of the acceptability of the intervention. Participants were also encouraged to write out their responses to the open-ended questions after the interview if they had additional thoughts or comments that they would like to contribute (see Textbox 1). Responses were recorded as field notes by the group moderator in a secured Microsoft Word document.

**Open-ended questions for in-person interviews.**

- Did you experience any physical or emotional discomfort during the virtual reality (VR) session – specifically, nausea or anxiety?
- Is there anything you would drastically change about the virtual environment in which we met – for example: colors, virtual items, or general surroundings?
- Do you feel there were any distractions preventing you from fully participating in the session?
- Would you like to see more opportunities to interact with each other, for example, through games or the ability to share digital media (photos, music, etc.)?
- Is there anything else you would like to share about the experience you just had?

### Description of Intervention

**Figure 1:**
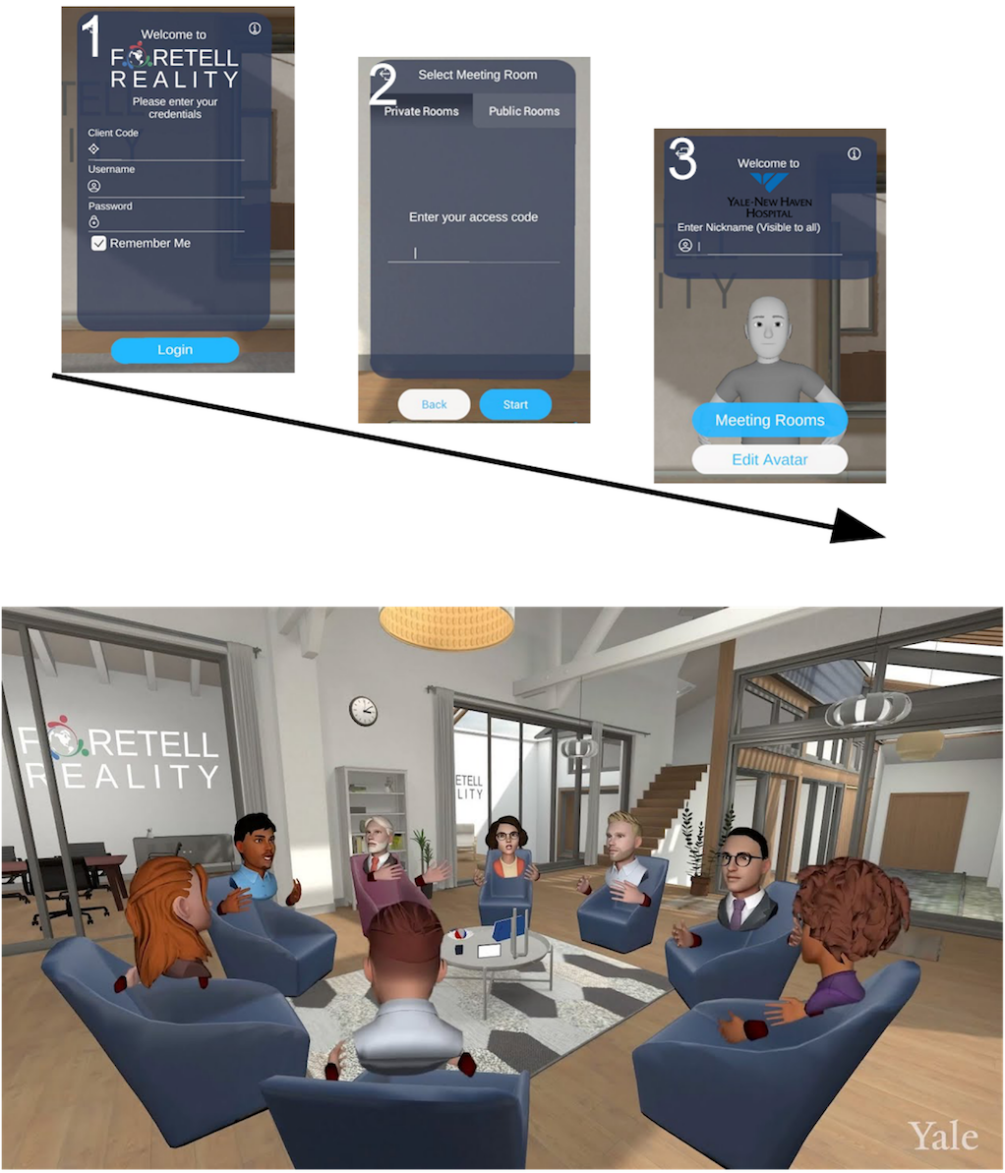
Sign-in process and example of the immersive therapeutic space.

Foretell Reality is a social VR platform that leverages VR technologies to bring people together for meaningful interaction in a virtual space. After a simple and secure sign-in process, invitees share the same immersive environment, as depicted in Figure 1. Participation can occur from anywhere in the world with an internet connection. The environment used in our intervention is that of a therapeutic space with participants seated in a circle (Figure 1). Though not tested as part of our pilot, optional activities directed by facilitators such as simple games (eg, “catch the ball”), drawing in 3 dimensions, joint viewing of 360 videos and pictures, presentation of documents, meditation, and physical exercise (eg, stretching or chair yoga) are available [30]

Seated in a circle, users feel a sense of social presence in VR. This is achieved through the tracked body movements and hand gestures of users’ customized 3D avatars, as well as spatial audio communications. Users can mask their voices and choose nicknames that allow for complete anonymity, which can help remove barriers to vulnerability. The platform is HIPAA compliant, does not store any identifiable personal information, is protected by individual login usernames and passwords, and requires special access codes to enter private meetings.

Working with Foretell Reality allowed us full control and customization of the features we chose to include in our support groups and provided us close support and training for our professional personnel and patients. For example, to ensure a calming and familiar environment where patients could focus on the session at hand, a room was created to resemble the Adolescents and Young Adults Lounge at Yale New Haven Hospital with minimal distractions. We specifically requested that features such as the ability to pass a ball around the room and move away from the seated area were turned off. Limiting movement around the room ensured a low likelihood of introducing experiences that could result in motion sickness.

### Study Procedure

Participants enrolled in this study were assigned to a support group on a first come first serve basis, assuming they met enrollment criteria. Groups were run one at a time with subsequent groups only enrolling after the previous group completed all sessions. Upon enrollment, patients were briefly oriented to the headset and software and given their sign in information.

During a support group session, 4 adolescent and young adult patients with cancer used the Meta Quest 1 (Meta Platforms, Inc; previously Oculus Quest 1) VR headset—a portable, low cost (US $300 manufacturer’s suggested retail price) piece of hardware running Foretell Studios (Foretell Studios) software—to participate in 6 virtual, professionally facilitated support groups. Participants received brief training on hardware and software use when the hardware was initially distributed prior to the start of the support groups. Participants also received a brief operating manual. The rules of engagement (Textbox 2) were reviewed with each participant prior to their enrollment.

**Rules of engagement.**

- Share only what you feel comfortable sharing.
- What is discussed in group, stays in group (unless there are concerns about safety).
- Be respectful/everyone should have an opportunity to share/speak.

Each support group session included 4 patients, a facilitator, and a research assistant (or the PI). The patients and facilitator were represented by a customized avatar, each with a chosen name displayed floating above their head. The research assistant or PI did not have a visible avatar, but participants were made aware that study personnel were potentially observing each session via VR headset or desktop computer. Participants were informed that the VR headsets could be used in the patients’ homes, inpatient hospital room, or any other space that had an active Wi-Fi signal and was convenient, private, and desirable for the patient. The 4 participants in each group were curated by the facilitator of the group in order to ensure the grouping of appropriate ages and development levels. In total, 4 separate groups of 4 participants each were assembled for a total of 16 participants and a goal of 22 individual support group sessions (the number of sessions was increased from 4 to 6 after string feedback from group 1). Each session lasted 45-60 minutes and was scheduled with a goal of occurring once weekly for 6 weeks. Sessions were postponed if fewer than 2 participants were available to participate during any 1 scheduled session.

Support groups were facilitated by an appropriately credentialed and trained clinical psychologist, psychiatrist, or social worker or their trained designees who reviewed the “Rules of engagement” (Textbox 2) prior to each session.

Once a group completed their last session, headset disinfection was done following methods of those described in a paper published by Roberts et al [31] using isopropyl alcohol wipes on all high-touch surfaces.

### Safety

With the introduction of any new technology, it is important to consider all possible malfunctions and emergent scenarios. All participants were given 2 phone numbers at the beginning of each session: the direct phone number to the facilitator and the direct phone number to the observing or on-call research assistant or PI. Additionally, the facilitator had an emergency phone number for each participant, as well as the physical location from which the participant was participating. While the hardware and software being used in this trial has been extensively tested and is commercially available, we developed troubleshooting and emergency intervention (Multimedia Appendix 1) protocols that were given to all participants. The greatest concern that we felt had to be addressed was that in an immersive, remote setting where patients are addressing topics with significant risk of inducing posttraumatic stress disorder, depression, and anxiety, it was important that they could find support immediately and at any time. In addition, given the new technologies we were dealing with, it was important that tech support was also available at any point during the session.

### Statistical Analysis

#### Quantitative Analysis

Descriptive statistics were obtained for each of the categorical variables pertaining to participant characteristics, including age, type of cancer diagnosis, documented gender identity within the electronic medical record (EMR), documented race and ethnicity within the EMR, and documented insurance status within the EMR. Attendance data were collected for each of the participants within each support group to evaluate attendance rates. Participants’ scores for the Connor-Davidson Resilience Scale and each of the PROMIS domains (positive affect, anxiety, and depression) before and after the support group will be compared using a 2-tailed *t* test with 95% CI. All statistical analyses will be completed using Stata (version 16.0; StataCorp).

#### Qualitative Analysis

Participant responses of the open-ended questions during the brief in-person interviews were recorded as field notes by the research coordinator and organized in a secure Word document. Using a grounded theory approach [32], we will use the rigorous and accelerated data reduction (RADaR) technique [33], which is used to organize, reduce, and analyze qualitative data. The RADaR technique is a rigorous, systematic approach to qualitative analysis that provides an expedited and user-friendly way of organizing, reducing, coding, and analyzing qualitative data. The RADaR technique is often used to manage smaller pilot projects that use grounded theory [27].

## Results

### Enrollment

Between November 5, 2019, and July 8, 2021, a total of 20 participants aged 17-20 years were approached to be a part of this study, and 16 agreed to participate. Reasons cited for not agreeing to participate included the lack of time and lack of interest. No patients expressed concerns around using the VR headset itself.

The enrollment of group 1 was completed in November 2019, in February 2020 for group 2, in August 2020 for group 3, and in May 2021 for group 4. Of note, groups 3 and 4 were enrolled amid the COVID-19 pandemic, and group 2 was recruited just before and subsequently ran during the pandemic. This resulted in prolonged times between group enrollments due to risks of COVID-19 being spread by unnecessarily returning to clinic for study consents, orientation, and headset exchange.

### Sample Demographics

The patients who enrolled had a median age of 19 (IQR 17-20) years. Overall, 62% (n=10) were male and 31% (n=5) were female, with 1 (6%) patient who identified as transgender female. In total, 5 (31%) participants identified as Hispanic, 1 (6%) identified as non-Hispanic Asian, 3 (19%) identified as non-Hispanic Black, 6 (38%) participants identified as non-Hispanic White, and 1 (6%) identified as other, as described in Table 1.

**Table 1:** Participant demographics and characteristics (n=16).

Characteristics		Value
Age (years), median (IQR)		19 (17-20)
**Documented gender identity in EMR^a^, n (%)**		
	Male	10 (62)
	Female	5 (31)
	Transgender female	1 (6)
**Documented race and ethnicity in EMR, n (%)**		
	Hispanic (all races)	5 (31)
	Non-Hispanic Asian	1 (6)
	Non-Hispanic Black	3 (19)
	Non-Hispanic White	6 (38)
	Other	1 (6.)
**Documented insurance status in EMR, n (%)**		
	Medicaid	7 (44)
	Private	9 (56)
**Type of cancer or disorder, n (%)**		
	Hematologic malignancies or bone marrow failure	8 (50)
	Solid tumor	6 (38)
	Central nervous system tumor	2 (12)

^a^EMR: electronic medical record.

Participation rates for each of the groups are shown below in Table 2. Group 1 was only scheduled for 4 total sessions, and future groups were increased to 6 sessions due to overwhelming requests from the first group to increase the total number of sessions. Of note, 1 of the participants in group 2 (g2p2) had a lengthy hospitalization shortly after they consented to this study. Given the inability of the hospital internet bandwidth or hot spot to support the system, they were unable to participate in any of the sessions—this hurdle was later remedied with a long-term evolution hot spot. Additionally, part of group 2’s and all of group 3’s and group 4’s sessions occurred during the COVID-19 pandemic. Further, 45% (10/22) of sessions needed to be postponed by a week or more, but only 18% (4/22) by more than 2 weeks.

Defining retention as attending any of the first 3 sessions in combination with any of the last 3 sessions, all but 2 patients met the criteria (g3p2 and g4p4) for a retention rate of 86.7% (SD 0.35%). Patients g3p2 and g4p4 described scheduling issues and the lack of interest as reasons for attrition.

**Table 2:** Participation rates.

Group and participant ID			Sessions attended, n (%)
**Group 1 (n=4 sessions)**			
	g1p1	4 (100)	
	g1p2	3 (75)	
	g1p3	4 (100)	
	g1p4	4 (100)	
**Group 2 (n=6 sessions)**			
	g2p1	5 (83)	
	g2p3	3 (50)	
	g2p4	5 (83)	
**Group 3 (n=6 sessions)**			
	g3p1	4 (67)	
	g3p2	1 (17)	
	g3p3	5 (83)	
	g3p4	6 (100)	
**Group 4 (n=6 sessions)**			
	g4p1	4 (67)	
	g4p2	5 (83)	
	g4p3	3 (50)	
	g4p4	2 (33)	

## Discussion

### Principal Findings

This protocol review describes the implementation of social VR support groups for a rare disease population with substantial participation (72.8%, SD 25.7%) and retention rates (86.7%, SD 0.35%). These rates compare favorably to historical data describing outpatient mental health attrition rates ranging widely with a range of 25% to 75% in children and adolescents [34]. Prior to the launch of our VR-based approach, and despite expressing a desire to participate in support groups, our adolescent and young adult cancer social worker was unable to convince any significant number of patients to return to the hospital for in-person groups. With the VR group option, however, we had a generally successful experience with manageable hurdles to implementation. No sessions needed to be canceled due to purely technical issues. We had no patients refuse to participate due to consent concerns and enthusiastic patients and families expressed a desire for expanded groups to include more sessions as well as requests for opportunities for caregivers to participate in their own groups.

The process of protocol development and review, however, have revealed an academic and health care system unprepared for the incoming wave of digital health interventions and subsequent implementation. PRCs and IRBs are designed to be led by experts and laypersons versed in the proposed interventions and familiar with the expected timelines and processes required for a smooth launch. These committees are required and helpful check points prior to the launch of any clinical trial at the Yale Center for Clinical Investigation. As this trial involved cancer patients with cancer, these committees have had substantial experience reviewing, recommending amendments to, and launching clinical trials involving early and late phase pharmaceutical trials. A supportive care trial involving cutting edge digital technologies requires an entirely different set of experience and skills. As a result, approval for what is a relatively benign intervention took more than 9 months, multiple protocol revisions, expansion, and the need to incorporate multiple outside entities including technology and psychologic experts to ensure scientific merit and safety. Due to this experience, we recommend an ad hoc digital health subcommittee be put in place at institutes interested in pursuing such digital interventions.

At a minimum, we propose that a digital health intervention protocol review and human protection committee would incorporate the expertise of digital privacy experts, software or hardware developers, and psychologists versed in the effects of immersive reality. To expedite a review, this committee would act as a first line, prior to submission to the PRC and subsequently the IRB. Given the inherent risks of health data (including biometric data) collection during the use of digital interventions, the committee would ensure appropriate security protocols are in place to ensure HIPAA compliance. With the increasing disconnects in the speed between technologic advancement and academic processes, they would additionally ensure that the technologies being studied are the most up to date and not soon to be outdated. Finally, this committee would be cognizant of the significant potential psychologic effects of immersive technologies and be prepared to address them.

Additional institutional barriers experienced included in-hospital networking protocols that limited the use of certain security approaches used by Foretell’s software. Subsequently, the hospital wireless network was not a viable solution. Fortunately, we were able to secure a reliable (long-term evolution) connection via mobile hot spot router, which was used for connection when study participants were in the hospital.

These barriers revealed challenges that may arise in attempting to implement similar interventions in more rural areas and counties other than the United States. While network speed demands are not high (we found no difficulty running sessions with upload and download speeds in the 10-30 Mbps range), reliability and stability are important. We anticipate that other high-income countries with a robust and reliable internet infrastructure should be successful in similar approaches. The expansion of this study will be looking specifically at non–English-speaking populations, rural populations, and populations defined as low socioeconomic status.

A noted limitation to this study is that participants were told that they may be observed by a team member other than the facilitator (PI or research assistant) during their sessions. This may have influenced how a participant behaved or what was said during a session. To mitigate this concern, observers were made invisible during the sessions and there was no indication of whether the observer was present or not. Given that the social worker themselves was present, visible, and participating in avatar form in every session, we suspect that any bias that may have been introduced by knowing an additional observer may be there was likely mitigated.

A final barrier to safe implementation of the above protocol was safe disinfection of the headsets themselves. This study coincided with the COVID-19 pandemic, so finding a safe way to continue offering support groups was essential. Concurrent with this project, we conducted an examination of VR equipment disinfection protocols, the results of which appear in the *Journal of Medical Internet Research* [31]. Despite initial excitement over ultraviolet disinfection, isopropyl alcohol or quaternary ammonium products were ultimately found to be most effective. Future studies should examine best uses and methodologies for an ultraviolet disinfection approach.

### Conclusions

Here we have presented an in-depth review of the creation and implementation of a novel, social VR-based intervention for an underserved, rare disease group. We have demonstrated feasibility through participation and retention and described pain points in implementation including the need for appropriate review committees, intrahospital network infrastructure, and disinfection needs.

These results and experiences have given us confidence to expand the trial to additional centers throughout the United States. The greater number of patients in more diverse populations with cancer will allow us to better assess feasibility in non–English-speaking populations, rural populations, and populations with low socioeconomic status. The expanded numbers will also offer greater power to identify effects on fear, anxiety, depression, resilience, and feelings of connectedness. In addition, we have started groups with the Yale Gender clinic, enrolling transgender and gender-diverse patients to examine the intervention’s effects on such measures as gender dysphoria, psychological flexibility, and self-esteem.

As immersive technologies and opportunities for innovative telehealth models emerge, it will be important to find their appropriate places within the clinical landscape. Populations with rare diseases seeking peer-based social support appear to be a viable and enthusiastic group with which to start.

## Appendix

Multimedia Appendix 1 Emergency procedures.

## Abbreviations

EMR: electronic medical record
HIPAA: Health Insurance Portability and Accountability Act
IRB: institutional review board
PI: principal investigator
PRC: Protocol Review Committee
PROMIS: Patient-Reported Outcomes Measurement Information System
RADaR: rigorous and accelerated data reduction
VR: virtual reality
