# Supplementary material for: Use of Immersive Virtual Reality Spaces to Engage Adolescent and Young Adult Patients With Cancer in Therapist-Guided Support Groups: Protocol for a Pre-Post Study
Source: JMIR Res Protoc. 2023 Nov 9;12:e48761. doi: 10.2196/48761 (PMC10667982; doi:10.2196/48761)
Supplement: Multimedia Appendix 1 [file resprot_v12i1e48761_app1.docx]

# Multimedia Appendix 1. Emergency procedures.

- - - 1. In the event that any group member finds themselves disconnected from an active session or the hardware suddenly fails, the below steps should be taken in the following order:
         1. The participant is to call the group facilitator to assure them that he/she is safe, but are having technical issues
         2. The participant is to refer to troubleshooting steps given at study consent
         3. If these steps do not fix the issue, the participant will call the observing research assistant or PI for assistance
      2. In the event that any group member finds themselves with physical discomfort, including nausea, headache, or confusion, the below steps should be taken in the following order:
         1. The participant is to exit the software and remove his/her headset
         2. The participant is to call the group facilitator to assure them that he/she is safe, but is having physical discomfort
         3. The participant should sit out for the rest of the session and report the discomfort to the study research assistant, PI, or facilitator within 24 hours
      3. In the event that any group member finds him/herself in psychologic or psychiatric crisis, including nervous breakdown, suicidal, or homicidal ideation, the below steps should be taken in the following order:
         1. The participant is to exit the software and remove his/her headset
         2. The participant is to call the group facilitator to inform them of the feelings he/she is having
         3. The facilitator will then use his/her professional judgement to instruct the member what to do next
